# Conventional versus Digital Impressions for Full Arch Screw-Retained Maxillary Rehabilitations: A Randomized Clinical Trial

**DOI:** 10.3390/ijerph16050829

**Published:** 2019-03-07

**Authors:** Paolo Cappare, Gianpaolo Sannino, Margherita Minoli, Pietro Montemezzi, Francesco Ferrini

**Affiliations:** 1 Dental School, Vita-Salute University and IRCCS San Raffaele, 20132 Milan, Italy; minolimargherita@gmail.com (M.M.); ferrini.f@gmail.com (F.F.); 2 Department of Dentistry, IRCCS San Raffaele Hospital, 20132 Milan, Italy; gianpaolosannino@gmail.com; 3 Oral Surgery Specialization School, Vita-Salute University and IRCCS San Raffaele, 20132 Milan, Italy; m.montemezzi@libero.it

**Keywords:** digital impression, digital workflow, full-arch rehabilitations, implant-prosthodontic restorations, immediate loading, crestal bone loss, implant survival, implant success

## Abstract

*Background*: The objective of this study was to compare conventional versus digital impressions for Full-Arch maxillary rehabilitations. *Methods*: Patients selected for this study were treated with full-arch screw-retained rehabilitations supported by six immediately loaded dental implants. Patients have been scheduled randomly into control (conventional impression group, CIG) and test (digital impression group, DIG) groups respectively for a fully conventional workflow and a fully digital workflow. In both groups, within 24 h, temporary prostheses were delivered. Four months after the implant positioning, the two groups dealt with the fabrication of definitive restorations: conventional pick-up was performed in the control group, and definitive digital impressions were carried out in the test group. The time involved following these two procedures was recorded. Patients underwent intraoral digital radiographs to evaluate the accuracy of the framework-implant connection, check for the presence of voids at the bar-implant connection and measure bone level. Criteria used to assess success at the prosthetic level were the occurrence of prosthetic maintenance, the absence of fractures of the acrylic resin superstructure and voids. *Results*: A total of 50 patients received immediately loaded prostheses supported by six implants (total 300 implants). A fixture and prosthetic survival rate of 100% was observed. All digital X-ray examinations revealed a bar-implant connection accuracy and no voids. Differences that were not statistically significant (*p* > 0.05) in marginal bone loss were found between control and test groups. Significantly less time was spent to perform digital impression procedure (*p* < 0.05). *Conclusions*: Clinical and radiological results of the test group advocate a satisfactory accuracy and predictability of the intraoral scanner (IOS) to be a reliable alternative in clinical practice for implant full-arch rehabilitations and suggest fabrication of definitive restorations with a successful marginal fit precision.

## 1. Introduction

The process of osseointegrated implant-supported rehabilitations of edentulous, or partially edentulous, jaws is considered a challenge for both clinicians and patients.

Developments in scientific research and existing evidence base helped the consensus for rehabilitations combining axial [1] and non-axial implants placed with immediate loading techniques to overcome the limitations of bone quality, especially in the maxillary and above all in the posterior areas, as an alternative to more invasive procedures as bone grafting [2,3] that can be associated with an increase of complications, morbidity, high costs and therefore decrease of their acceptance by patients [4].

The benefits of this protocol fit the actual expectancies of patients including mini-invasivity, immediate functional and esthetic outcome [1] and a reduction of the overall treatment time [5].

The traditional workflow for implant prosthetic rehabilitations have been chosen in clinical practice for a long time as the leading technique, although it is a procedure that requires several manual manufacturing steps, as well as skilled dental technicians and impression materials, are prone to undergo dimensional variations [6]. 

The accuracy of the impression, that is considered according to a review of the literature [7] the main factor influencing the structures’ fit, is affected by impression material, impression technique, implant angulation, the number of implants. An optimal fit of the implant-fixed prosthesis is required for its long-term success [8]. Any incorrect framework may lead to mechanical complications as screw loosening or fracture [8] and biological complications, which could compromise the bone–implant interface [9] and also the homogeneity of the occlusal load.

Even though no technique has yet been identified as the gold standard, intraoral digital impressions can be considered a reliable alternative for fixed implant prosthetic restorations [10,11,12]. The elaboration of a digital approach has been proven to be even more accurate [13] and efficient [14] than conventional materials. Since the digital impressions are instantly sent and saved/stocked electronically for the fabrication of definitive prosthetic restorations [15], thus enhancing efficiency of the workflow, the IOS has proved the decrease of margin of error caused by traditional impression taking [13] and cast production [16] methods. Several published studies have examined digital impression techniques in implant dentistry [11,12,16,17,18,19,20,21]: Papaspyridakos et al. [10] concluded in a comparative study that the use of intraoral scanners for full-arch implant rehabilitations is significantly more accurate than conventional impressions. An in vitro study of 2019 [22] comparing impression techniques for dental implants concluded that in a clinical situation with less than 3 implants conventional impression is more accurate, while in cases of 4 implants the IOS has a superior accuracy.

Furthermore, from a systematic review [23] evaluating the advantages of digital technologies for the manufacturing of implant-supported rehabilitations, it emerged the increased efficiency of CAD/CAM systems compared to conventional fabrication procedures.

The aim of this clinical study was to evaluate the most accurate impression technique for Full-Arch rehabilitations screwed over six implants, comparing conventional and digital impression workflows.

## 2. Materials and Methods

### 2.1. Patient Selection

From March 2016 to December 2016, patients were randomly selected for this clinical study performed at the Department of Dentistry at San Raffaele Hospital, Milan, Italy.

The following inclusion criteria were chosen: patients in good general health of any race and gender and acceptable oral hygiene, physically and psychologically able to be submitted to implant surgery and restorative procedures (ASA 1, ASA 2), edentulous in the upper arch or with unfavourable long-term prognosis teeth, sufficient bone for 6 implants and adequate stability for immediate function. 

All procedures performed in this study involving human participants were in accordance with the ethical standards of the institutional and/or national research committee and with the 1964 Helsinki declaration and its later amendments or comparable ethical standards. The ethical committee number of approval is CE/INT/10/2015.

### 2.2. Pretreatment

The diagnosis and the treatment plan of each patient were established clinically, through oral examination with inspection and palpation, and radiographically (preoperative panoramic radiograph and computed tomography CT) in order to point out bone morphology and its volumetric sizes, evaluating vertical and horizontal resorptions. For the maxillary post-extraction sockets, bone quality and quantity were accurately assessed following the Joudzbalys classification [24] system. To all patients were provided written informed consent for immediate implant loading and for digital impression procedure. Subsequently, they were given 2 g amoxicillin (Zimox, Pfizer Italia, Latina, Italy) one hour before the procedure. 

### 2.3. Surgical Procedure

In the edentulous maxillary patients, incisions were performed on the alveolar ridge and followed by releasing incision bilaterally. The presence of keratinized tissue on both margins of the horizontal incision has been obtained in order to facilitate the suture and the achievement of the biological seal of the implant-soft tissue interface [25,26]. Dissection was carried out with respect to the cleavage planes and full thickness submucoperiosteal flaps were reflected. Implant site was prepared according to the surgical procedure suggested by the manufacturer instructions (CSR implant system, Sweden & Martina, Due Carrare, Padova, Italy), with standard drills following the palatal wall as guide, and the apical portion of implant site was prepared at least 4 mm beyond the apex. The coronal margin of the implant was located 0.5 mm apically to the buccal level of the bone crest. The implant (CSR, Sweden & Martina, Due Carrare, Padova, Italy) had a machined neck of 0.8 mm and a bevel of 0.3 mm for the platform switching technique, the coronal segment presents a tapered morphology, the central portion is cylindrical and the apex is tapered, with a round shape and 4 incisions [27,28]. In each patient, 6 implants were inserted in the maxilla. Firstly the 4 posterior implants were placed axially, if needed the two distals were tilted to avoid the maxillary sinus and were inserted near and in parallel to the anterior sinus wall. In these last cases, tilting didn’t overcome 30 degrees. When required, crestal levelling was achieved with a bone scraper for an omogenous subcrestal positioning in the posterior arches and bone recontouring was performed to level for tilted implants. Afterwards, the 2 anterior implants were positioned axially. Implants had a final insertion torque of at least 40 N cm and when in presence of soft bone high primary stability was obtained through the application of underpreparation techniques.

To compensate for reduced parallelism between implants, when needed, pre-angled 17 degrees abutments (Sweden & Martina, Due Carrare, Padova, Italy) were positioned.

These angles were selected so as to guide the prosthetic screw access holes into an occlusal or palatal position. 

Lastly, soft tissues were sutured without tension with 4-0 nonresorbable sutures.

### 2.4. Post-Surgical Instructions

Following surgery, patients received indications to prevent from brushing and any trauma to the surgical site. 

As postoperative care, nonsteroidal anti-inflammatory drugs (Brufen 600 mg, Abbott Laboratories, Chicago, IL, USA) and chlorhexidine digluconate 0.2% mouthwash were prescribed for 2 weeks together with 1 g of amoxicillin twice a day (Zimox, Pfizer Italia, Latina, Italy) for one week after the surgical procedure. 

Furthermore, patients were required to follow a cold diet for the first day and a soft diet (avoiding hard and crunchy foods) for the first 2 months of healing.

### 2.5. Prosthetic Protocol

Patients have been scheduled randomly into control (conventional impression group, CIG) and test (digital impression group, DIG) groups respectively for a fully conventional workflow and a fully digital workflow.

Randomization processes occurred by lots in closed envelopes and were peformed by a blinded operator.

The vertical dimension was established through the recording of several facial reference marks before surgery.

In both groups, within 24 h, screw-retained acrylic resin temporary prostheses were delivered and positioned at a torque controlled wrench at 15 N cm. Immediately after the surgical procedure, the interim restorations have been properly intraorally relined with autopolymerizing polyurethane resin (Voco, Fort Mill, SC, USA). 

Static, dynamic occlusion and centric contacts on all crown surfaces were carefully verified and modified where needed through the use of an articulating paper (40 μm Bausch Articulating Paper, Nashua, NH, USA). Following the immediate provisional prosthetic protocol [29], centric and lateral contacts were limited to the intercanine area and also passive fitting was checked.

Temporary resin (Fermit, Ivoclar Vivadent, Naturno, Bolzano, Italy) was utilized to cover screw access holes.

Subsequently, after a 4-month healing time of soft and hard tissues, the two groups dealt with two different approaches for the definitive prosthesis manufacturing: conventional pick-up versus digital impressions. 

From the control group (CIG), Full Arch prosthetic rehabilitations were manufactured using traditional impression techniques. The impression material used was gypsum (Éclair Class II, Ultima, Angers, FRANCE).

Impression transfers were screwed over the fixtures and splinted together using orthodontic wire and composite resin, that has been gradually polymerized in order to reduce and avoid its shrinkage. After obtaining the impression, it was necessary to attach the implant analogs to the copings and then realize a stone model to replicate the exact position of the implant in the cast. 

In the test group (DIG), a digital scanner was utilized to fabricate the definitive prostheses. The scan body, replacing the traditional impression coping, enables an intraoral digital scanner to accurately capture the implant fixture. Scan bodies (for CSR, Sweden & Martina, Due Carrare, Padova, Italy) were splinted together as in the traditional technique, applied for splinting transfers.

The intraoral scanner used in the present study was a Carestream CS 3600 (Version 3.1.0 Acquisition Software, Carestream Dental LLC, Atlanta, GA, USA).

The Carestream system transforms into a virtual volume the three-dimensional geometry of dental arches using the principle of structured light. 

A scanning stitching strategy [30] was carried out by the same experienced investigator and was applied to DIG group for the splinted scan bodies.

IOS light source was formerly kept parallel to the occlusal plane and started from the distal implant of the upper left quadrant and moved toward the anterior implant of the upper controlateral quadrant (right side); it was then moved back to the distal implant of the left side, tilting it toward the palatal side; the occlusal plane was crossed toward the buccal side and the camera was moved again from the starting scanning point of the left side to the anterior implant of the right side trying to keep it orthogonal to the occlusal plane. The image was then inspected and missing regions were filled by fast passages of the camera over the related areas.

**Figure 1:**
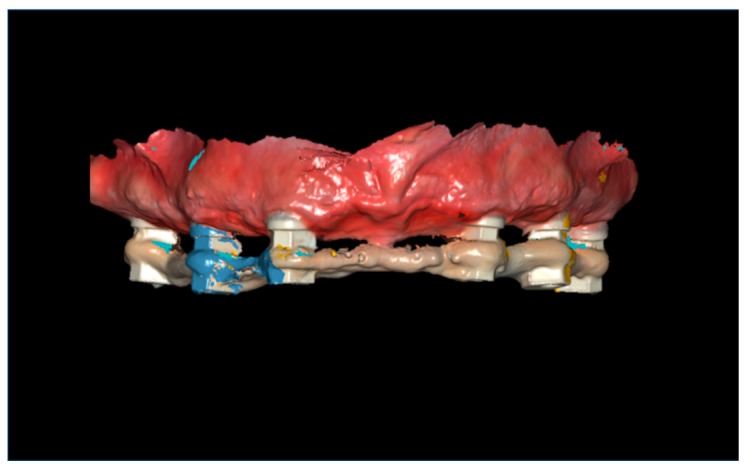
Full-arch digital scan with splinted scan bodies.

The same scanning procedure was performed with the other half, from the anterior implant of the upper left quadrant to the distal implant of the upper controlateral quadrant (right side). The software automatically applied a stitching algorithm in order to merge the two halves, based on the area between the two anterior implants, shared by both the separate scans. (Figure 1).

**Figure 2:**
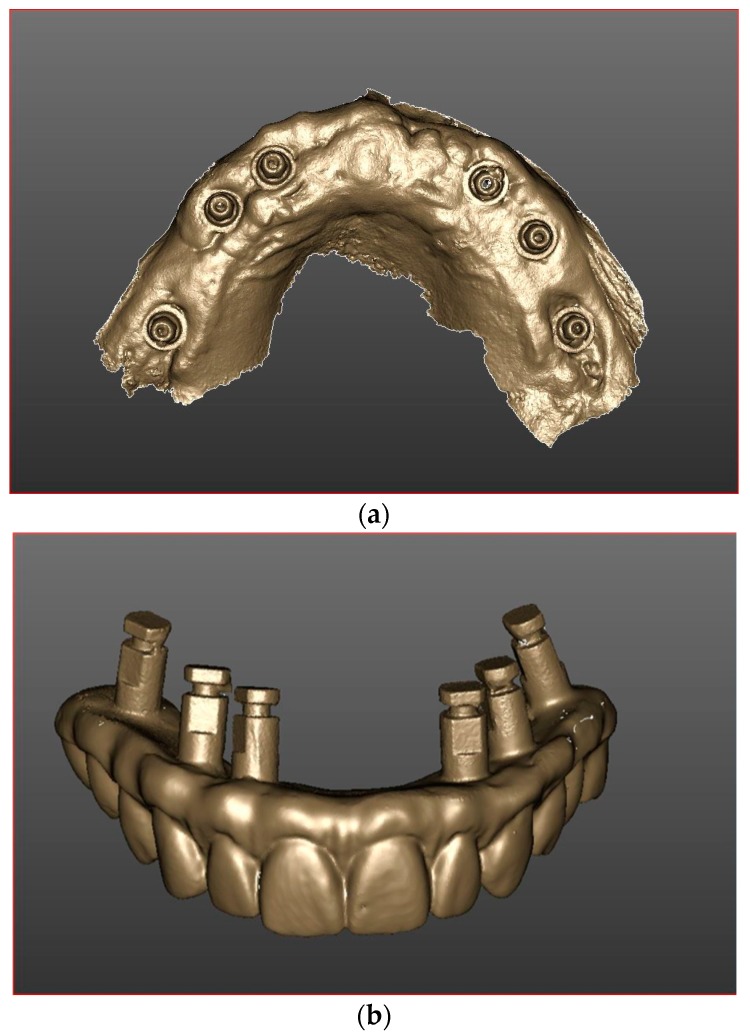
(**a**) Intraoral scanning of soft tissues after a 4-month healing time. (**b**). Scan of the temporary prosthesis with analogs in position.

**Figure 3:**
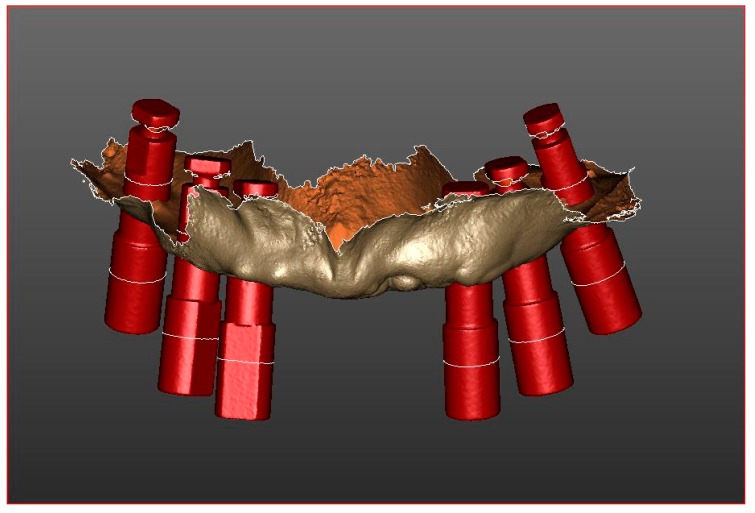
Matching of soft tissues, scan bodies and virtual models of related components scans.

Furthermore, after the acquisition of the splinted scan bodies, the operator has performed a scan of soft tissues (Figure 2a). and an intraorally scan of the temporary prosthesis with analogs in position (Figure 2b). Subsequently, the scans have been matched for each patient (Figure 3).

The opposing arch was scanned with the same procedure, followed by a scan of the buccal aspect of the patient’s dentition in maximum intercuspidation. The resulting 3D scans were then exported in the standard tessellation format (.stl). 

**Figure 4:**
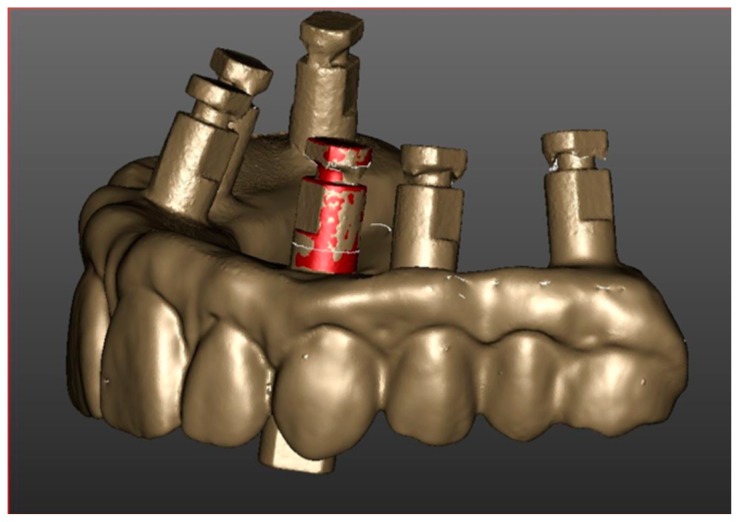
Precision match of intra and extraoral scan.

The virtual model of the temporary prosthesis, attached to implant analogs, is then also acquired extraorally through a laboratory scanner (Neway, Open Technologies, Rezzato, Italy) to verify the accuracy of the intraoral scanning [17,31] (Figure 4). In cases in which a difference between the intra and extraoral scanning was noticed, the restoration would be realized using the extraoral scan as a reference.

In both groups, the time needed for impression procedures was recorded in seconds.

All the scans of the scanner were uploaded to the laboratory and returned after post-processing. The virtual images were evaluated for accuracy of detail and correct occlusal relationship.

**Figure 5:**
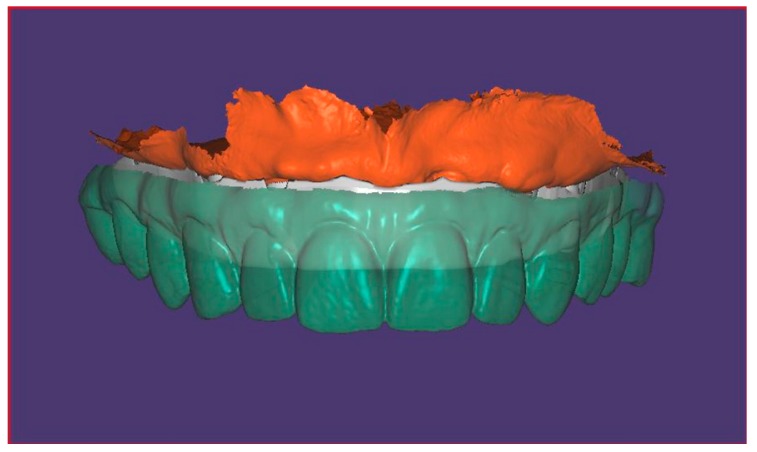
Virtual models of the framework and of the superstructure.

Once the virtual model is created with the dental implant in position, virtual digital creation of framework and restorations can be designed through the CAD software (Exocad software, Darmstadt, Germany) (Figure 5).

**Figure 6:**
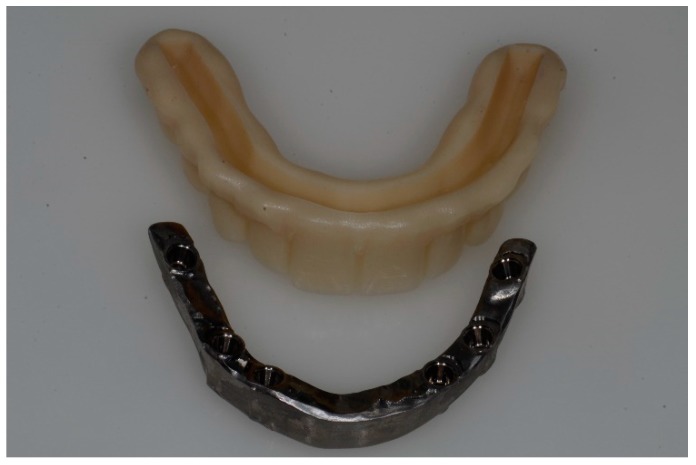
CAM output of the framework and of the suprastructure.

As the definitive frameworks are being milled from titanium, the rapid prototype model is simultaneously sent to the dental laboratory for use in fabricating the definitive restoration (Figure 6). 

**Figure 7:**
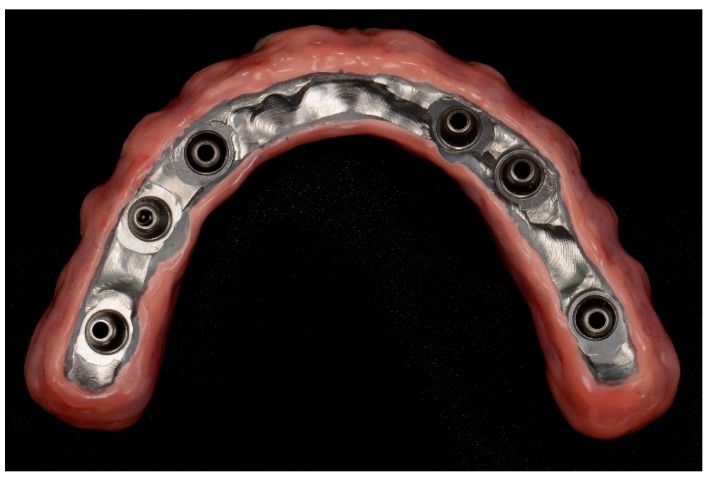
Prosthesis finalization in the laboratory.

In none of the two groups the framework needed to be cut nor soldered. The prosthesis, set up for the esthetic trial placement, constituted by a framework and a suprastructure of esthetic material, has been realized on the basis of the temporary prosthesis, but refined in esthetics, function and in relation to soft tissues. After the esthetic and functional evaluation, the restoration is finalized, over the mesostructure the esthetic material has been added for the rebuilding of teeth with composite (NACERA HYBRID, DOCERAM Medical Ceramics GmbH, Dortmund, Germany) and soft tissues. Prosthesis was then cemented to the titanium mesostructure with a dual cure cement (RelyX Unicem, 3M, St. Paul, MN, USA). In both groups, 50 definitive prostheses were manufactured using metal frameworks in order to increase the strength and rigidity of restorations (Figure 7).

**Figure 8:**
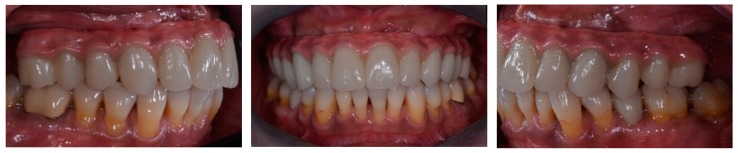
Definitive intraorally screw-retained restorations.

All prostheses were positioned and screwed onto dental implants (Figure 8). Sheffield test was carried out to check the precision of framework. 

The marginal fit of final prosthetic frameworks screwed onto the implants was checked by intraoral digital radiographic examination in both groups. Articulating paper (Bausch Articulating Paper, Nashua, NH, USA) was used to check the occlusion and adjust it, if necessary. In particular, static occlusion consisted of central contacts established on all masticatory units and dynamic occlusion included canine/premolar guidance, regardless of the opposite arch settings. Screw access holes were covered with temporary resin (Fermit, Ivoclar Vivadent, Naturno, Bolzano, Italy).

Outcomes of impression techniques were evaluated using the following clinical acceptance criteria: (1) accurate imprinting of implant areas; (2) no voids on the occlusal, buccal, and lingual sides; and (3) proper reproduction from the vestibule up to the mucogingival junction [12]. Impressions not meeting the criteria underwent retakes for conventional impressions or rescans/further scans.

Total treatment time and retakes/rescans required to meet acceptance criteria were evaluated to prove the efficacy of the two impression techniques. Treatment time (minutes:seconds) was represented by the time required to obtain an acceptable impression, in accordance with criteria. When necessary, impression retakes (conventional impressions) and rescans of missing areas (digital impressions) were registered as extra working time and additional events. 

### 2.6. Follow-Up

Follow-up visits were carried out by a dental hygienist at 3, 6, 12 and 24 months after implant insertion. Success criteria for implant survival were depicted by the presence of implant stability and absence of radiolucency around implant sites and for peri-implant soft-tissue by mucosal suppuration, bleeding, plaque index, pain and probing pocket depth. The criteria used to assess success at the prosthetic level were the occurrence of prosthetic maintenance, the absence of fractures of the acrylic resin superstructure and voids. Implant survival was defined as the absence of implant mobility, swelling, or pain in the surgical site at the time of examination. 

Implant success was established as implant survival plus marginal bone loss of less than 1.5 mm after 1 year of loading and no more than 0.2 mm of loss between each follow-up appointment.

### 2.7. Radiographic Examination

**Figure 9:**
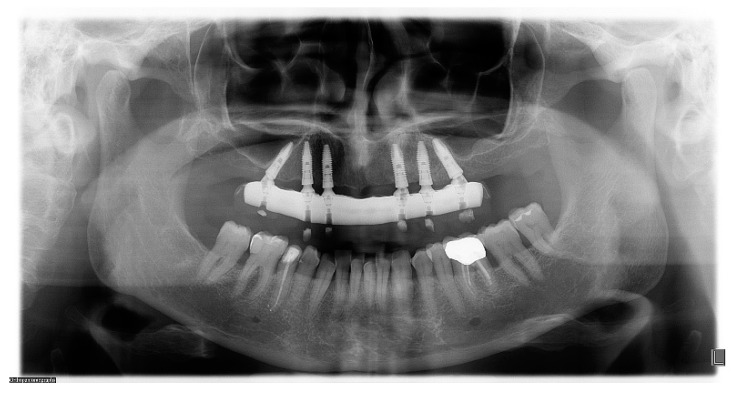
Panoramic radiograph at follow-up.

Radiographic evaluations were performed using panoramic radiographs obtained immediately after surgery and at each follow-up visit (Figure 9). 

The primary criteria followed to assess the impression outcomes was the absence of voids at the bar–implant connection.

The marginal fit precision of final prosthetic frameworks of both groups, screwed-retained onto the implants, were checked by intraoral digital radiographic examination (DIGORA 2.5 software Soredex, Tuusula, Finland) immediately after placement. Bone level measurements were performed on the mesial and distal aspect of each implant, using the implant-abutment junction as a reference point. To adjust the dimensional distortion and enlargement on the radiographs, the actual sizes of the implants were compared to the measured implant dimensions on the radiograph. A blinded radiologist measured variations in marginal bone height over time and presence of voids at the bar-implant connection: he marked the reference points and measured lines on the screen interactively (the numeric value of measurements was reported by specific software (DIGORA 2.5 software Soredex, Tuusula, Finland). The implant height (a known dimension) was used for calibration. Mesial, distal and mean bone loss were calculated in the maxilla, and the resulting data were reported.

All measurements were performed by a blinded operator.

### 2.8. Statistical Analysis

A dedicated software (SPSS 11.5.0, SPSS, Chicago, IL, USA) was used for all statistical analyses. Bone level measurements were reported as means ± standard deviations at 6, 12 and 24 months. Time needed for digital and conventional procedures was measured in seconds and reported as means ± standard deviations. Time spent and bone loss around the 6 implants was compared within group and between groups by means of the Student t-test at a significance level of *p* = 0.05.

To compare CIG and DIG in terms of treatment time and number of retakes/rescans, the Wilcoxon signed-rank test was used. *p* values at <0.05 were considered statistically significant.

## 3. Results

Fifty patients were treated with immediately loaded Full Arch prostheses supported by 6 implants (in total 300 implants). Implant dimensions were reported in Table 1.

The mean age of the patients was 64.4 years, with a range between 48 and 72 years.

Eleven patients were excluded from the study, seven of them for systemic diseases and four because they presented active infection in post-extraction sockets planned for implant placement. Other exclusion criteria were smoking more than 15 cigarettes per day and bruxism habits. 

Twenty-five patients have been treated through a fully digital workflow and 25 through a fully conventional workflow.

In no case a difference was seen between the intra and extraoral scanning.

A total of 50 definitive prostheses were manufactured using metal frameworks to increase the strength and rigidity of restorations. No dental implant drop out occurred. All prostheses were screwed onto the dental implants and intraoral digital X-ray examinations revealed a bar-implant connection accuracy. The fixture survival rate was 100% for all positioned implants. None of the 50 fixed prostheses were lost during the observation period, representing a prosthetic survival rate of 100%.

In the test group, all prostheses were screwed onto the dental implants and no void at the bar–implant connection was observed. 

The analysis of procedure time revealed that the digital impression procedure took less time than did the conventional procedure (Table 2), and the difference was statistically significant (*p* < 0.05). Digital impressions needed more rescans than conventional impression needed retakes; however, the time for a rescan was far less than that for a retake (Table 2).

At the 24-month evaluation a mean peri-implant crestal bone loss averaged 1.07 ± 0.66 mm in CIG and of 1.11 ± 0.54 mm in DIG (Table 3). Not statistically significant differences were found between CIG and DIG (*p* > 0.05).

## 4. Discussion

In all full-arch rehabilitations definitive prosthesis screwed onto six implants revealed clinically and radiographically a very accurate bar-implant connection with absence of voids. 

The key finding of this clinical study, considering implant success as the main evaluated parameter, was that homogeneity in the two groups was observed: no statistical differences between DIG and CIG could be marked. In both groups the maintenance of bone levels over time can be explained by the new conical implant-abutment connection of the positioned implants that seems to be resistant against bacterial microleakage [28].

The outcomes of this prospective study, in accordance with previous studies [12,17], clearly show how the impression accuracy and laboratory performance of digital and conventional approaches is similar, affects the prognoses of full-arch rehabilitations and proves excellent clinical results. The results of a few previous studies even demonstrated a superior accuracy, in terms of trueness and precision [15] of digital impressions for full-arch restorations compared to conventionals [10,32,33,34,35]. 

Since the recent introduction in the market of CS 3600 intraoral scanner, only a few articles [36,37,38,39] have been published in literature evaluating its accuracy. Imburgia et al. [39] conducted a study to compare the ability of four different IOS: CS 3600 proved the highest trueness both in partially with 3 implants and fully with 6 implants edentulous models while all four scanners offered similar results in precision. From another comparative study [38] evaluating the finish line distinctness (FLD) and accuracy (FLA) in 7 intraoral scanners versus conventional impression it has emerged that CS 3600 presented the highest FLA together with Trios. 

In addition, the test group (DIG) of this clinical study demonstrated a reduced impression time, and improved patient comfort and acceptance. The findings of two cohort studies, aimed at accomplishing a cost/time analysis, even revealed that the digital workflow was approximately threefold more efficient in term of time [40] and 20% less expensive in term of cost [41] than the traditional protocol. Among several studies that have discussed patients’ perceptions and treatment comfort towards impression techniques, Yuzbasioglu et al. [42] reported a subjects’ preference of computer-aided impressions and furthermore the digital impression took approximately 248.48 ± 23.48 s versus conventional impression 605.38 ± 23.66 s.

Two other DIG advantages have been pointed out in this study: the risks related to the traditional material distortions were overcome and the 3D previsualization allowed a real-time check of the scanning correctness.

Indeed, a criticity of the traditional workflow for implant-supported full-arch rehabilitations, due to the intrinsic limits of impression material elasticity, is the incapability to reproduce excessive disparallelisms in complex cases, up to causing the retaking of an impression or leading to the impossibility of coping the implant position with reasonable accuracy [34]. 

CAD/CAM systems were formerly introduced in dentistry for single elements over thirty years ago, and advancements in technology has enlarged its field of application up to manufacture complex multi-unit rehabilitations [43]. A few articles in peer-reviewed journals evaluated the use of IOS for full-arches [10,15,32,44,45]. Among the aforementioned studies, even though there is no consensus yet regarding the acceptable 3D misfit, Amin et al. [10] in an in vitro study indicated a mean 3D deviation of 167.93 μm for full-arch conventional impressions, greatly differing from the mean 3D deviations of Omnicam and True Definition both below 50 μm. From another in vitro study conducted by Abdel-Azim et al. [32] it emerged a higher accuracy of full-arch digital impressions with a measured marginal discrepancy of 63.1 μm compared to the conventional impressions marginal discrepancy of 135.1 μm.

Another strength of this study is characterized by the fact that only a small number of articles [17,46] have used marginal bone loss in maxilla as a term of comparison between a fully digital versus conventional approach and both studies reported in accordance with this present prospective study an implant success rate of 100% and no statistically prosthesis survival differences between the 2 groups.

The results of the present clinical protocol were in contrast with a previous review evaluating conventional impressions accuracy as compared to optical impressions [47] since it still appeared to be the leading technique for implant-supported full arches long-span rehabilitations, as implant-supported full arches.

Several theses, related to errors registration during IOS scanning, have been reported in the literature to support the superiority of conventional impressions: local deviations [16], decreasing accuracy for error accumulation due to the distance between fixtures on posterior teeth of opposite quadrants [48,49,50], misleading factors as limited intraoral space, saliva, compliance of the patient [51], scanning strategy [52,53,54], software version. The impact of software and scanning strategy on the accuracy of the scan should be tested for each IOS model, even though the results of a study [30] comparing two different approaches of full-arch scans showed a better precision of the Stitching technique in contrast with the No Stitching, pointing out a smaller standard deviation and having a relevant aspect from a clinical perspective. 

A limitation of the present study could be that the digital protocol can be considered an operator-sensitive technique [48]. Gimenez et al. reported that the operator influenced the accuracy of measurements. An operating learning curve may be required to develop the appropriate skills.

Lee et al. [55] investigated the difficulty level and operator’s perception of computer-aided versus traditional implant impressions among dental students and experienced clinicians. The student group scored a conventional impression mean difficulty level significantly higher, while there wasn’t a significant difference in the difficulty level of the two groups (30.6 for the student group and 36.5 for the clinician group). As to operator’s preference, digital impressions are easier for inexperienced clinicians (67%).

## 5. Conclusions

The test group of this study advocate a satisfactory accuracy and predictability of the IOS to be a reliable alternative in clinical practice to the conventional workflow for implant full-arch rehabilitations. The accuracy of CAD/CAM systems has shown to be compatible with conventional impressions.

On equal terms of the two approaches, the digital workflow seems to be a valid choice for full arch rehabilitations due to the less invasive option for patients and its time saving.

However, further clinical studies are necessary to assess the accuracy and efficiency of the digital workflow for full-arch restorations.

## Figures and Tables

**Table 1:** Implant diameters and lengths.

MAXILLA *n* = 300			
Total *n* = 300		length 13 mm	length 15 mm
	diameter 4.2 mm	13	30
	diameter 3.8 mm	214	43

**Table 2:** Time needed (minutes) for conventional and digital bone loss values (mean ± SD) for tilted and upright implants of both groups (CIG = conventional impression group; DIG = digital impression group).

Parameter	CIG	DIG	*p* Value
Procedure time	16:45 ± 4:49	8:59 ± 2:46	<0.05
Additional time	06:02 ± 2:01	2:34 ± 1:02	<0.05
No. of retakes	2	7	

**Table 3:** Crestal bone loss values (mean ± SD) for tilted and upright implants of both groups (CIG = conventional impression group; DIG = digital impression group) (CIG *n* = implant = 150; DIG *n* = implant = 150).

Bone Loss	IMPLANTS	
	CIG	DIG
6 months (mm)	1.03 ± 0.32	0.99 ± 0.48
12 months (mm)	1.04 ± 0.56	1.08 ± 0.52
24 months (mm)	1.07 ± 0.66	1.11 ± 0.54
